# cAMP-dependent protein kinase (PKA) complexes probed by complementary differential scanning fluorimetry and ion mobility–mass spectrometry

**DOI:** 10.1042/BCJ20160648

**Published:** 2016-09-27

**Authors:** Dominic P. Byrne, Matthias Vonderach, Samantha Ferries, Philip J. Brownridge, Claire E. Eyers, Patrick A. Eyers

**Affiliations:** Department of Biochemistry, Institute of Integrative Biology, University of Liverpool, Crown Street, Liverpool L69 7ZB, U.K.

**Keywords:** complex, inhibitor, ion mobility, mass spectrometry, protein kinase A, protein structure

## Abstract

cAMP-dependent protein kinase (PKA) is an archetypal biological signaling module and a model for understanding the regulation of protein kinases. In the present study, we combine biochemistry with differential scanning fluorimetry (DSF) and ion mobility–mass spectrometry (IM–MS) to evaluate effects of phosphorylation and structure on the ligand binding, dynamics and stability of components of heteromeric PKA protein complexes *in vitro*. We uncover dynamic, conformationally distinct populations of the PKA catalytic subunit with distinct structural stability and susceptibility to the physiological protein inhibitor PKI. Native MS of reconstituted PKA R_2_C_2_ holoenzymes reveals variable subunit stoichiometry and holoenzyme ablation by PKI binding. Finally, we find that although a ‘kinase-dead’ PKA catalytic domain cannot bind to ATP in solution, it interacts with several prominent chemical kinase inhibitors. These data demonstrate the combined power of IM–MS and DSF to probe PKA dynamics and regulation, techniques that can be employed to evaluate other protein-ligand complexes, with broad implications for cellular signaling.

## Introduction

Protein phosphorylation is a reversible post-translational modification (PTM) catalyzed by the protein kinase-mediated enzymatic transfer of ATP γ-phosphate to an appropriate side chain in a substrate [1]. A plethora of techniques, including X-ray crystallography, protein nuclear magnetic resonance (NMR), enzyme kinetics and (more recently) molecular dynamics, differential scanning fluorimetry (DSF) and mass spectrometry (MS), have been exploited to dissect enzymology, supramolecular structure, allosteric co-operativity and nucleotide/ligand-binding propensity within protein kinase complexes [2–8]. Concomitantly, many members of the protein kinase superfamily have become important therapeutic targets, in part due to their inherent druggability [9], but also because they play rate-limiting roles in many human diseases, including inflammation, heart disease and cancer [10]. Protein kinases are ubiquitous and evolutionary related, possessing canonical amino acid motifs that are positioned at the catalytic interface, trapping divalent metal cofactors and positioning protein substrate directly adjacent to the ATP phosphoryl donor, which is thought to bind prior to substrate in the catalytic cycle [11,12]. This conserved mode of ligand binding implies that the development of specific probe compounds to interrogate protein kinase catalytic function is a challenge, and much remains to be learnt about the chemical and protein ligand sensitivity of ‘open’ and ‘closed’ (lying between ‘inactive’ and ‘active’) conformational states that exist within dynamic populations of kinase signaling complexes [13]. Although considerable progress has been made in the synthesis and exploitation of kinase-specific ligands, some of which employ allosteric (non-ATP)-binding modes, the vast majority of clinically approved compounds interact with the nucleotide-binding site. Consequently, the cellular effects of ATP-competitive drugs have the potential for nonspecific polypharmacology [14] caused by unpredictable interactions with different kinase conformers and a variety of ‘off-target’ proteins.

The discovery of cAMP-dependent protein kinase (PKA) in 1968 established the importance of second-messenger-regulated signaling complexes in signaling [15]. The founding members of the ‘AGC’ family of Ser/Thr protein kinases are the three human PKA catalytic (C) subunits (PKAc), two cGMP-regulated kinases (PKG) and nine PKC family members, all of which share common structural features [16,17] that have been employed for design and profiling of protein kinase inhibitors [18,19]. However, PKAc is generally regarded as a drug ‘anti-target’ due to its central role in human physiology. This model protein kinase has also been central to X-ray (and later NMR) studies that have established it as a structural and enzymatic paradigm for benchmarking the entire human kinome [20]. This came about in part because components of the PKA complex can be overexpressed, (de)phosphorylated, myristoylated and affinity purified for experimental exploitation *in vivo* [21–23], and assembled into multivalent signaling complexes for analysis *in vitro* [24]. The regulation of PKA is unusually complex, involving a variety of PTMs. In common with other AGC kinases, the catalytic activity of PKAc is dynamically regulated *in cis* through flanking elements of the polypeptide and enzymatic post-translational (auto)phosphorylation at multiple conserved sites [25,26]. In addition, N-terminal PKAc myristoylation has been demonstrated in human cells [27], where this modification is reported to drive PKA holoenzyme complexes to the plasma membrane [28]. This is in addition to well-characterized effects on intrinsic stability (but not catalysis) *in vitro* [29]. PKAc enzyme activity is controlled by a plethora of partner proteins that make up complex physiological signaling complexes [3,30]. In the absence of cAMP, catalytic activity and subcellular location of PKAc are tightly constrained by binding to functionally nonredundant regulatory (R) subunits, which generate physiological combinations of R and C assemblies. The ‘textbook’ (catalytically inactive) R_2_C_2_ PKA holoenzyme originally purified from tissues [31,32] is thought to represent a physiologically relevant complex in human cells [22]. PKA is activated by localized fluxes in cAMP concentration mediated through opposing effects of families of adenylate cyclases and nucleotide phosphodiesterases [16]. Co-operative cAMP binding at two sites per R subunit unleashes PKAc, facilitating the phosphorylation of physiological substrates containing a minimal Arg-Arg-X-Ser PKA consensus motif [33]. In the PKA signaling module, further layers of regulation are encoded by intracellular targeting through A-kinase anchoring proteins (AKAPs), which interact mainly with RII subunits [30]. PKA is further regulated through potent pseudosubstrate inhibition of PKAc by three isoforms of the heat-stable inhibitor protein of PKA, termed PKI [34–36], whose physiological roles are slowly being uncovered in vertebrates [37,38]. Inhibitory sub-nanomolar affinity binding of PKI to PKAc is also thought to expose a canonical nuclear export signal in PKI, which may redistribute the (inhibited) complex between different intracellular compartments [39]. Whether RII and PKI binding to PKAc is mutually exclusive remains unknown.

An appreciation that protein kinases are inefficient enzymes has strengthened the idea that they act in cells as regulated macromolecular switches, whose structural dynamics and cross-domain communication enable transition between low- and high-activity conformations. X-ray analysis confirms that PKA holoenzmes are subject to allostery. Moreover, different C- and R-subunits can co-assemble to generate distinct quaternary complexes, although their composition and stoichiometry are potentially susceptible to artifacts of crystallization. Importantly, intrinsic co-operativity within the C-subunit exists [40], so that ligand binding is sensed conformationally, potentially coupling ATP site occupancy with substrate dynamics during the catalytic cycle [41]. Classes of small-molecule or protein ligands might therefore exhibit differential effects on PKA signaling, potentially through distinct binding modes, as noted for several kinase and inhibitor combinations [42,43]. For PKAc, the discovery of regulatory hydrophobic ‘spines’, whose assembly and disassembly enable communication within the bi-lobal kinase domain [44], enables ligand binding to have far-reaching consequences, including allosteric coupling across networks or communities of amino acids [45,46].

In the present study, we exploit the complementary analytical approaches of solution DSF [47] and gas-phase ion mobility–mass spectrometry (IM–MS) [48] to characterize highly purified and re-assembled components of the PKA complex and explore the effects of ligand binding. Thermostability assays (TSAs), such as DSF, have recently demonstrated great promise in quantifying the (de)stabilizing effects of protein–ligand interactions both *in vitro* [49] and *in cellulo* [50], whereas ‘native’ MS and IM–MS are increasingly being exploited to interrogate the structure and dynamics of protein complexes [48], and analysis of different classes of small-molecule ligands [7,51,52].

DSF of PKAc reveals discrimination of compounds and ligands with affinity for catalytically ‘active’ and ‘inactive’ PKAc variants. In addition, we provide IM-MS-based evidence for conformation-specific binding of PKI to PKAc, and demonstrate native stoichiometric and sub-stoichiometric PKA complexes. Finally, a dramatic ablation of R_2_C_2_ tetrameric complexes by PKI, with preferential formation of a stable PKA:PKI heterodimer, is established for the first time. Together, our work represents a new approach to study macromolecular PKA complexes that can be readily extended to other kinase complexes and ligands *in vitro*.

## Experimental

### Protein expression and purification

All proteins analyzed were produced in BL21 (DE3) pLysS *Escherichia coli* cells (Novagen) with expression induced with 0.5 mM IPTG for 18 h at 18°C. Murine PKI (α-isoform) and PKAcα1 (nonmyristoylatable) were cloned into the pET-30 Ek/LIC vector (Novagen) and purified as N-terminal His6-tag fusion proteins by immobilized metal affinity chromatography and size-exclusion chromatography (SEC) using a HiLoad 16/600 Superdex 200 column (GE Healthcare) equilibrated in 50 mM Tris–HCl, pH 7.4, 100 mM NaCl, 10% (v/v) glycerol and 1 mM DTT. PKAc K72H and R133A point mutants were generated by PCR site-directed mutagenesis [21], expressed, and purified as above. N-terminal 6His-tagged RIIα subunit was expressed as described previously [53]. Glutathione-*S*-transferase (GST) tagged λ protein phosphatase (λPP) was cloned into pGEX-6P-1 and purified with Glutathione-Sepharose 4B (GE Healthcare). Secondary structure compositions of wild-type (WT) and mutant PKAc proteins (0.6 mg/ml) were analyzed by circular dichroism (CD) in the far UV range (180–260 nm) using a Jasco 1100 CD spectrometer with a path length of 0.1 cm, following exchange into 10 mM sodium phosphate (pH 7.4) and 25 mM NaF.

### Phosphopeptide enrichment and LC–MS/MS

Protein samples were reduced and alkylated prior to overnight digestion with trypsin (2% w/w) and subsequent phosphopeptide enrichment using Titansphere Phos-TiO_2_ spin tips (GLSciences) with minor modification of the manufacturer's instructions (see Supplementary Material). Tryptic peptides were analyzed by LC–MS/MS before and after TiO_2_-based phosphopeptide enrichment. nLC–electrospray ionization (nESI)-MS/MS analysis was performed using either an Orbitrap Fusion Tribrid mass spectrometer (ThermoScientific) attached to an Ultimate 3000 nanoLC system (Dionex) or an AmaZon ETD ion trap (Bruker Daltonics) arranged in-line with a nanoAcquity n-UHPLC system (Waters). .mgf files were searched using MASCOT (version 2.1) against the *E. coli* IPI database (downloaded 24 March 2015) with the complete sequence of 6His-tagged PKAc included. Carbamidomethylation of Cys was set as a fixed modification; phosphorylation of Ser and Thr, oxidation of Met and deamidation of Asn and Gln were set as variable modifications. The tandem mass spectra for all identified phosphopeptides were interrogated manually.

### Dephosphorylation of PKAc with GST-λPP and PKA kinase assay

PKA (60 µg; ∼13 µM, 100 µl final volume in 50 mM NH_4_OAc buffer) was incubated at 37°C with 100 ng of λPP in the presence of 1 mM Mn(OAc)_2_ for 2 h. The reaction was stopped by buffer exchange into 50 mM NH_4_OAc. For kinase assays, 1 ng of purified PKAc, or R133A PKAc was assayed in the presence and absence of recombinant full-length His-tagged PKI using a Caliper EZ Reader II enzyme assay platform in 25 mM Hepes (pH 7.4), 1 mM ATP, 5 mM MgCl_2_, 1 mM DTT and 5 µM fluorescent 5-Fam-Leu-Arg-Arg-Ala-Ser-Leu-Gly-CONH_2_ (Kemptide). Activity (kinetic mode) was reported as % peptide phosphorylation after ∼10 cycles.

### DSF assays

Thermal shift assays were performed with a StepOnePlus Real-Time PCR machine (Life Technologies) using Sypro-Orange dye (Invitrogen) and thermal ramping (0.3°C per minute between 25 and 94°C). All proteins were diluted to 5 μM in 50 mM Tris–HCl (pH 7.4) and 100 mM NaCl in the presence or absence of the indicated concentrations of ligand [final DMSO concentration 4% (v/v)]. Nucleotide, PKI peptide (amino acids 5–24) or kinase inhibitors (diluted from 10 mM DMSO stocks) were assayed in the presence or absence of the appropriate divalent cations, as described. Data were processed using the Boltzmann equation to generate sigmoidal denaturation curves, and average *T*_m_/Δ*T*_m_ values were calculated as recently described [47] using GraphPad Prism software.

### Detection of PKAc and PKA RIIα binding by SEC

Equimolar concentrations (∼2.5 μM) of catalytic (C; WT or K72H) and RIIα (regulatory) PKA subunits were preincubated at 4°C for 2 h and loaded onto a Superdex 200 10/300 GL column (GE Healthcare) equilibrated in 50 mM Tris–HCl (pH 7.4), 100 mM NaCl. Eluted fractions (0.5 ml) were collected, and complex formation was analyzed by SDS–PAGE and Coomassie blue staining. Binding was confirmed by co-elution of the C and RIIα subunits from the column.

### Ion mobility–MS and collisional activation

IM–MS analysis was performed on a Waters Synapt G2-S*i* instrument. All proteins were buffer exchanged into 50 mM NH_4_OAc (LC grade, Sigma) employing Amicon spin filter columns (10 kDa molecular cutoff). Typically, 1–3 µl of 2–5 µM sample was analyzed using borosilicate emitters (Thermo ES 387). Spraying voltage was adjusted to 1.1–1.8 kV and the sampling cone was 20 V. Pressure in the travelling wave (T-wave) ion mobility cell was 2.78 mbar (nitrogen); wave height was kept at 30 V and wave velocity at 750 m/s. For collision-induced unfolding (CIU) experiments, a single charge state was quadrupole isolated and subjected to collisional activation by applying CID activation in the ion trap of the instrument. The activation voltage was varied between 25 and 41 V. The ion mobility wave height was 40 V and the wave velocity was 650 m/s. Data were processed with MassLynx 4.1 and OriginPro 9.0 and used to generate contour plots of the intensity of ion populations as a function of collision cross section (CCS) and collision voltage. Noncovalent interactions were studied by mixing the corresponding components in 200 mM NH_4_OAc and equilibrating for at least 10 min at room temperature prior to MS analysis.

### CCS calibration and theoretical calculations

To experimentally determine CCS, the measured drift time through the T-wave mobility cell of β-lactoglobulin A (Sigma L7880), avidin (Sigma A9275), transthyretin (Sigma P1742), concanavalin A (Sigma C2010) and serum albumin (Sigma P7656) was calculated according to standard protocols [54]*.* The exact hard sphere scattering (EHSS) model implemented in the Mobcal software was used to calculate CCS values on the basis of X-ray structures [55].

## Results and discussion

### Analysis of purified recombinant proteins

**Figure 1.**
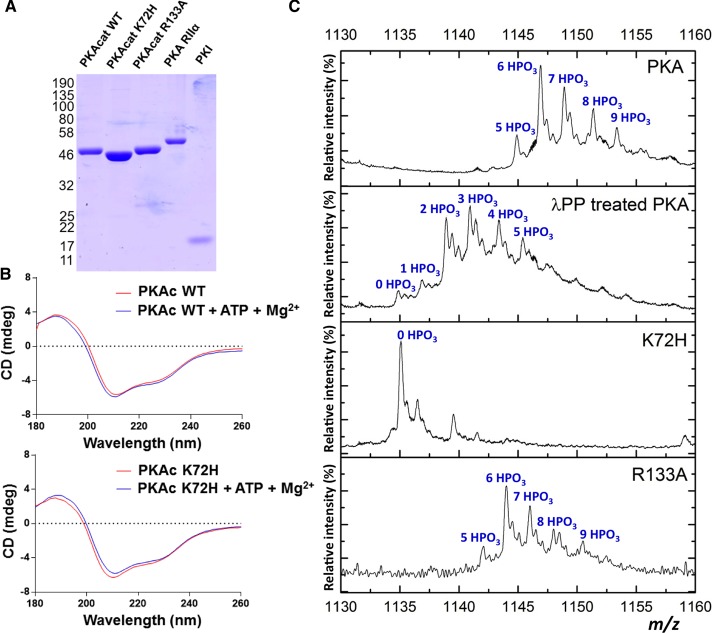
Analysis of purified recombinant proteins that make up the PKA signaling complex. (**A**) Coomassie blue staining of purified recombinant PKA proteins: 1.5 μg of PKA catalytic (WT, K72H or R133A proteins) and regulatory (RIIα subunits and 0.6 μg of PKI protein were analyzed by SDS–PAGE and proteins stained with Coomassie Blue). Coomassie staining of PKI is weak due to its small size and amino acid composition. (**B**) CD spectra demonstrating similar secondary structures of WT and K72H PKAc in the absence or presence of 1 mM ATP and 10 mM Mg^2+^ ions. The mean average of three replicate spectra of 0.6 mg/ml WT (red) and K72H (blue) PKAc in 10 mM sodium phosphate (pH 7.4), and 25 mM NaF, recorded in a 0.1 cm cell are shown. (**C**) ESI mass spectra of the 39+ charge state of intact recombinant PKAc WT (PKA) under denaturing conditions, in the absence (top) or presence of Mn^2+^-λ protein phosphatase (λPP), and for PKAc K72H and PKAc R133A. Peaks are annotated with the number of phosphate groups.

We expressed and purified to homogeneity a panel of proteins that can be used to reconstitute multimeric or holoenzyme (R_2_C_2_) PKA signaling complexes *in vitro*. We also generated mutations in the catalytic subunit that have previously been shown to selectively prevent autophosphorylation and abrogate catalytic activity [56] (‘kinase-dead’ K72H PKAc mutant) or that specifically reduce binding to the RIIα subunit and the heat-stable inhibitor of PKA (PKI), while sparing autophosphorylation and catalytic activity [21,57] (R133A PKAc mutant). As shown in Figure 1, these proteins were pure, and SEC and DSF (see below) suggested that all proteins were folded and monomeric. CD also confirmed similar secondary structures in both WT and K72H PKAc proteins when analyzed in the presence and absence of ATP and Mg^2+^ ions, the latter inducing no major changes in the spectra. A key goal of our study was to determine the effects of phosphorylation on PKA complex formation *in vitro*. Using peptide-based tandem MS analysis, we initially determined the sites of phosphorylation on PKAc proteins, confirming that it was highly phosphorylated on several known sites, including Ser10, the key T-loop (Thr197) and C-terminal tail motif (Ser338) sites, these being required to generate a stable, catalytically active PKAc [26,58,59]. Interestingly, our comprehensive sequence coverage revealed a total of 11 sites of phosphorylation in recombinant WT and 11 phosphosites on R133A PKAc, all of which were absent from K72H PKAc preparations (Table 1 and Supplementary Material), confirming that they are autophosphorylation sites.

**Table 1:** Sites of autophosphorylation on PKAc WT before and after treatment with λPP, and of PKAc R133A, identified by LC–MS/MS analysis of tryptic peptides

Sample	Sequence	Site
WT, R133A	*KGpSEQESVK	*Ser10
R133A	WEpTPSQNTAQLDQFDR	^†^Thr32
WT, R133A	WETPpSQNTAQLDQFDR	Ser34
WT, R133A	WETPSQNpTAQLDQFDR	^†^Thr37
WT	IKpTLGTGSFGR	Thr48
WT, R133A	IKpTLGTGpSFGR	Thr48, Ser53
WT, R133A	IKTLGpTGSFGR	Thr51
WT, R133A	*TLGTGpSFGR	*Ser53
WT, R133A	HKEpSGNHYAMK	^†^Ser65
WT, R133A	*IGRFpSEPHAR	*Ser139
WT, R133A	*TWpTLCGTPEYLAPEIILSK	*Thr197
WT	*FPpSHFSSDLK	*Ser259
WT, R133A	*VpSINEK	*Ser338

* Sites preserved following the treatment of WT PKAc with Mn^2+^–λPP.

† Indicates novel phosphosites.

### Phosphorylation regulates PKA conformation

To assess effects of phosphorylation on the structure and intrinsic stability of the PKA catalytic subunit, recombinant intact autophosphorylated PKAc was analyzed by MS before and after treatment with λPP (Figure 1). We observed an average of ∼7 autophosphorylation sites in WT PKAc (Figure 1A), out of a total of 14 sites of phosphorylation that had been mapped (Table 1; Supplementary Tables S1–S3 and Figure S1); these identified sites of modification include three nonphysiological autophosphorylated residues found within the 6His affinity tag, whose nonspecific modification by basophilic kinases was noted in previous work [60]. Importantly, of the 11 phosphosites observed in the core PKAc polypeptide, we mapped phosphorylation at Ser34, only previously reported in a single high-throughput screen, and discovered two potential novel sites of modification in the kinase N-lobe (Thr37 and Ser65; Supplementary Figure S1). Previous work has demonstrated that recombinant PKAc is rather resistant to dephosphorylation at the T-loop (Thr 197) and C-terminal motif (Ser 338) phosphorylation sites [61,62]. Consistently, incubation with λPP, using either Mg^2+^ or Mn^2+^ as the divalent cofactor, generated hypophosphorylated forms of PKAc with a reduced average of ∼4 (data not shown) and ∼3 phosphosites per molecule, respectively, with 6 phosphosites mapped by peptide-based tandem MS (Table 1, Figure 1B,C, and Supplementary Table S3). Even after extensive incubation with λPP, complete dephosphorylation could not be achieved, providing us with an opportunity to analyze the conformation of differentially phosphorylated preparations of PKAc by native IM–MS. Crucially, K72H was observed in a completely nonphosphorylated form, consistent with the peptide-based analysis, and R133A yielded an intact phosphorylation phosphoprotein profile almost identical to WT protein (Figure 1C).

**Figure 2.**
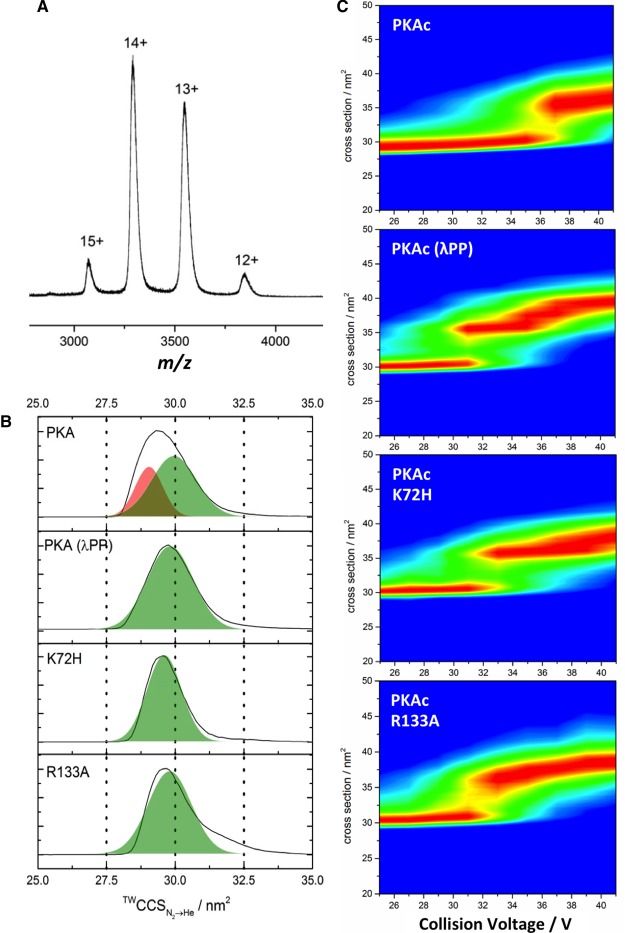
IM-MS of PKAc variants reveals conformationally distinct forms. (**A**) ESI mass spectrum of PKAc obtained under nondenaturing ‘native’ conditions. (**B**) ^TW^CCS_N2→He_ for the [M+13H]^13+^ form of untreated WT PKAc (PKA), PKAc following treatment with Mn^2+^-λ protein phosphatase (PKA λPP), and the K72H and R133A variants. Two overlapping conformations of PKA are indicated, the more extended of which (green) matches the CCSD of the other PKA species. (**C**; top to bottom) CIU profiles of PKAc WT, λPP-treated, K72H and R133A.

When ESI-MS was performed under nondenaturing (‘native’ solution) conditions, autophosphorylated PKAc was observed over an extremely narrow charge state distribution, predominantly yielding 13+ and 14+ ions (Figure 2A), indicative of highly ordered tertiary protein structure [63]. IM–MS was subsequently used to determine the CCS distributions and, following calibration of the T-wave ion mobility cell, a ^TW^CCS_N2→He_ value of 29.4 nm^2^ for the 12+/13+/14+ charge states of hyperphosphorylated PKAc was determined. As expected, charge-mediated unfolding of the 15+ species yielded multiple conformations with higher CCS values (Supplementary Figure S2). Comparison of CCS for PKAc before and after phosphatase treatment (Figure 2B,C) revealed phosphorylation-dependent effects on both the absolute ^TW^CCS_N2→He_ value and the half-height width of the CCS distribution (CCSD); CCS (Ω) increased by 1.5% following treatment with λPP, whereas CCSD demonstrably reduced from 2.2 nm^2^ (28.4–30.6 nm^2^) to 1.8 nm^2^ (28.8–30.6 nm^2^). Autophosphorylation thus promotes compaction of PKAc while increasing conformational dynamics. Interestingly, hyperphosphorylated PKAc also exhibited a noticeable asymmetry in the CCSD, not apparent upon phosphatase treatment. This phosphorylation-dependent increase in conformational space is perhaps unsurprising given the number of modified residues identified (Table 1) and the likelihood of nonstoichiometric phosphosite occupancy.

The K72H and R133A variants of PKAc were employed as comparators for further structural investigation of the PKA complex. Of particular interest for understanding the role of phosphorylation on PKAc conformation, stability and ligand-binding capabilities is the K72H PKAc mutant, given its inability to autophosphorylate (Table 1 and Figure 1). Although the potential folding status of this purified mutant cannot be assessed as a function of catalytic output, the near-identical CD spectra obtained for WT and K72H PKAc (Figure 1B) suggest similar secondary elements of structure in both proteins.

Gaussian fitting of the CCSDs determined by native IM–MS revealed two primary conformers for WT PKAc, with CCS values of 29.0 and 30.0 nm^2^, the larger of which overlaid with the predominant conformer apparent following phosphatase treatment (Figure 2B). To better understand the conformational stability of these PKAc variants, we performed a series of CIU experiments evaluating the effect on CCSD. By elevating the collision energy (CE) applied to the native-solution derived conformers (but maintaining it below that required for covalent bond dissociation), it is possible to gradually unfold a protein and thereby gain an understanding of its (gas-phase) stability and the structure of partially unfolded intermediates [64]. The CIU profile of hyperphosphorylated PKAc was markedly different from that of the phosphatase-treated form of PKAc (Figure 2C and Supplementary Figure S3). WT-untreated phospho-PKAc is significantly more stable, requiring higher CE to initiate unfolding. While λPP-treated PKA starts to undergo conformational change at as low as ∼31 V, WT PKAc is conformationally stable under these conditions, not undergoing significant unfolding until ∼36 V.

Akin to data obtained at low CE (Figure 2), the conformational space (CCSD) adopted by hyperphosphorylated PKAc at high CE (>40 V) is larger than the phosphatase-treated form. Interestingly, both K72H and R133A PKAc are more similar to λPP-treated than -untreated PKAc in their native state, with CCS values of 29.6 and 29.8 nm^2^, respectively. Furthermore, both exhibit CIU profiles more typical of the less stable, more open conformer of λPP-treated WT PKAc. That both PKA variants exhibit similar CCS values of lower conformational stability suggests that these structural effects are not primarily dependent on the extent of phosphorylation. In particular, the fact that the conformation of R133A PKAc overlays with the more elongated (open) conformer of WT PKAc led us to hypothesize differential roles for these two predominant PKAc configurations (see below).

**Figure 3.**
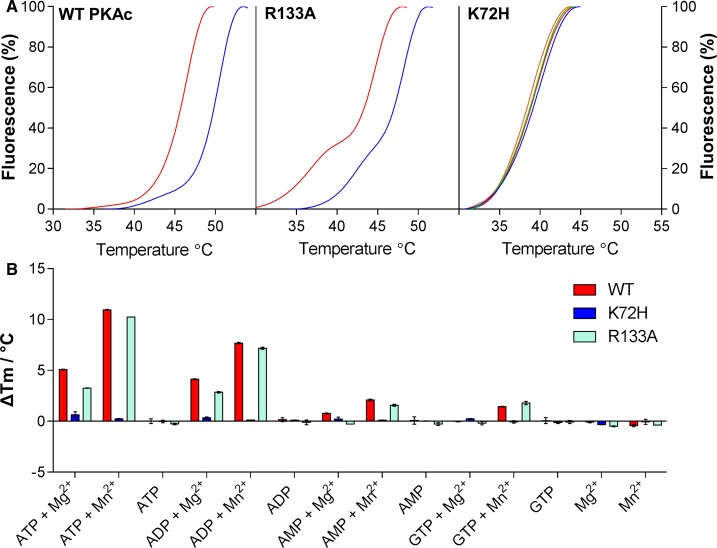
PKA binding of nucleotides is detectable by DSF. (**A**) TSA of PKA WT, K72H and R133A (5 μM) in the presence of 10 mM MgCl_2_ and 1 mM (blue), 2 mM (green) or 4 mM (orange) ATP; buffer control is in red. (**B**) Δ*T*_m_ for WT, K72H and R133A PKA upon nucleotide binding, as measured by DSF. Mean Δ*T*_m_ values ± SD (*n* = 2) were calculated by subtracting the control *T*_m_ value (buffer, no nucleotide) from the measured *T*_m_ value.

In agreement with the CIU profiles, WT PKAc was observed to have greater thermostability, as determined by DSF, than either R133A or K72H (Figure 3A). Although the *T*_m_ values at 50% were comparable for WT and R133A PKA (43–44°C), the thermal unfolding profiles were different, with R133A exhibiting a biphasic response and a 20% *T*_m_ value of ∼37°C (compared with ∼43°C for WT PKAc), suggesting rapid thermal unfolding of R133A to a more stable intermediate. In contrast, K72H PKAc was even less thermostable (*T*_m_ = 39°C), in agreement with previous literature findings [56].

### Small molecule and PKI binding to PKA is conformation-specific

In contrast with MS analysis, where electrospray ionization is suppressed by metal ions, a major strength of DSF is its ability to report nucleotide and metal interactions among active and inactive conformers present in a variety of (pseudo)kinase domain preparations [47]. We therefore exploited DSF to investigate the binding of nucleotides to PKAc. As expected [65,66], thermostabilization was readily observed for WT PKAc using a broad range of nucleotides (particularly ATP and ADP) in the presence of divalent cations (Figure 3B), and this was recapitulated with the R133A variant. In contrast with the increase in *T*_m_ observed for WT PKAc (Δ*T*_m_ = 4.24 ± 0.09°C) and R133A (Δ*T*_m_ = 3.49 ± 0.16°C) with 1 mM ATP and Mg^2+^, incubation of K72H PKAc (or a less stable K72R PKAc mutant, data not shown) with any combination of nucleotides or cations failed to promote any stabilization under these conditions (Δ*T*_m_ for K72H = −0.49 ± 0.04°C upon incubation with ATP/Mg^2+^ ions), even at very high concentrations (up to 4 mM) of ATP. We therefore believe that our assay reveals either a loss of nucleotide binding in the K72H mutant or an inability to detect thermal stabilization upon nucleotide binding. These findings contrast with previous work, which employed fluorescent ATP displacement (mant-ATP) and CD-based thermal unfolding in urea, to demonstrate that K72H C-subunit could bind to ATP and the small-molecule inhibitor H89, despite a detectable lack of phosphorylation or catalytic activity [56].

**Figure 4.**
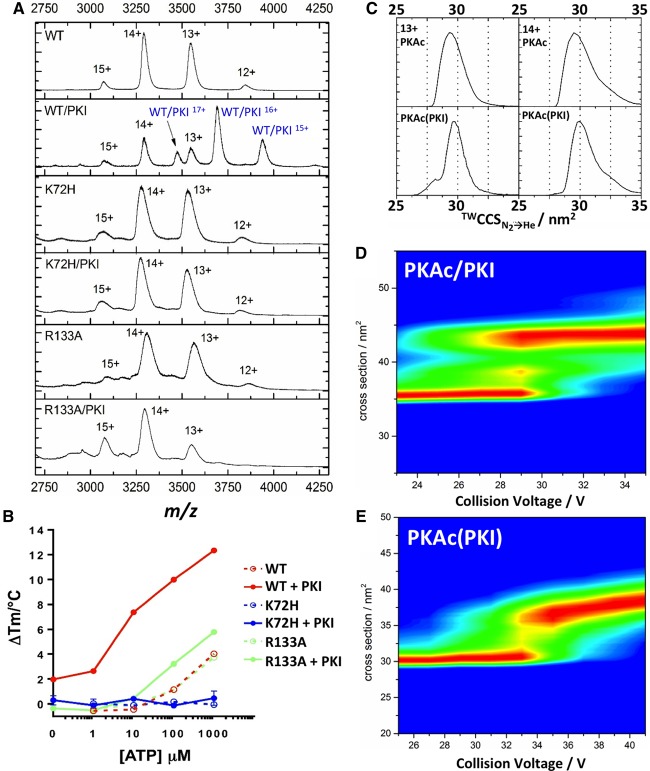
PKI protein binds stably to PKAc WT, but not K72H or R133A protein. (**A**) Native ESI mass spectra of PKAc WT, K72H and R133A, in the absence or presence of equimolar PKI. (**B**) TSA of WT, K72H and R133A PKAc proteins measured in the presence of the indicated concentration of ATP and 10 mM MgCl_2_ ± 10 μM PKI. Mean Δ*T*_m_ values ± SD (*n* = 2) are shown. (**C**) ^TW^CCS_N2→He_ for [M+13H]^13+^ and [M+14H]^14+^ forms of WT PKAc in the absence (top) or presence (bottom) of PKI. CCS distribution of non-PKI-bound form of PKAc is presented [PKA(PKI)]. (**D** and **E**) CIU profiles of PKAc upon the addition of PKI: (**D**) PKI-bound PKA (PKA/PKI) and (**E**) non-PKI-bound PKAc.

We next studied the effects of exogenous protein binding to PKAc, taking advantage of the resistance of PKI to denaturation at temperatures above 70°C, at which temperature PKAc is fully unfolded (Figure 3A). Binding of purified PKI inhibitor protein to all three PKAc proteins was assessed by both DSF and (IM-)MS. The high-affinity interaction of PKAc with PKI measured by DSF was confirmed by native MS with observation of the PKA:PKI heterodimer over multiple (15^+^–17^+^) charge states (Figure 4A). A Δ*T*_m_ value of +1.97 ± 0.04°C was determined for WT PKAc upon PKI binding (Figure 4B), while the IC_50_ value for inhibition of PKAc activity was in the sub nM range (Supplementary Figure S4), consistent with previous data measuring a Kd*app* of <0.2 nM [57]. However, neither K72H nor R133A formed a stable interaction with the inhibitor protein under the relatively mild conditions used for the MS analysis (Figure 4A), and both had negligible shifts in thermal stability as determined by DSF: 0.29 ± 0.37°C and −0.36 ± 0.01°C upon PKI addition for K72H and R133A variants, respectively (Figure 4B). Furthermore, R133A exhibited a nearly 200-fold higher IC_50_ value for inhibition by PKI (Supplementary Figure S4), confirming loss of enzyme inhibition despite unchanged autophosphorylation and ATP affinity. Interestingly, the binding of both PKI (pseudosubstrate inhibitor) and ATP/Mg (nucleotide) to the WT C-subunit was additive, or even synergistic, over a range of ATP concentrations (Figure 4B), confirming that DSF detects co-operative binding at two separate sites on PKAc, consistent with recent work [41].

To assess PKAc conformational dependence of PKI binding by IM–MS, it was necessary to look indirectly, by comparing the CCS (Ω) of the non-PKI-bound form of PKAc remaining in the presence of excess PKI (Ω_WT(PKI)_) with Ω of PKAc in the absence of PKI (Ω_WT_). The direct effects of PKI binding on the structure of PKAc could not be evaluated due to the innate presence of ligand and the resultant increase in complex size. IM–MS analysis of the non-PKI-binding form of PKAc revealed a small, but highly reproducible 1.4% increase in Ω, with a Ω_WT(PKI)_ value of 29.8 nm^2^ compared with Ω_WT_ of 29.4 nm^2^ (Figure 4). Interestingly, Ω_WT(PKI)_, that is the non PKI-bound form of PKAc, was essentially identical with that of the more elongated form of PKAc, suggesting conformer-specific binding of PKI to the more compact PKAc species. Our model of conformer-specific binding of PKI to PKAc is also supported by IM–MS analysis of PKA mutants; both K72H and R133A lack the ability to bind PKI (Figure 4) and both exhibit similar Ω values to WT(PKI).

Published crystal structures of PKAc lacking (PDB 13JH) or containing (PDB 1APM) a PKI-derived peptide (PKI 5-24) [67–69], allowed us to compare our experimentally observed differences in PKAc conformation with those from published X-ray structures. The EHSS model, implemented in Mobcal, was used to calculate Ω_EHSS_ for PKAc from PDB files. In agreement with our MS data, a decrease in PKAc Ω was observed upon PKI binding: Ω_EHSS_ (PKAc 11–350) = 33.1 nm^2^ and Ω_EHSS_ (PKAc 11–350 with PKI) = 32.2 nm^2^. Although Ω cannot be compared directly between our studies and the computed Ω_EHSS_ values from X-ray structures, primarily because the core PKA sequence is different in these two sets of experiments, the reduction in conformational space adopted by PKAc upon PKI binding is entirely consistent with our IM–MS data.

Interestingly, CIU profiles of the heterodimeric PKA–PKI complex (Figure 4D) revealed distinct partially unfolded intermediates, unlike the gradual elongation observed for the non-PKI-bound species. In contrast with the complex formed between PKAc and the nM kinase inhibitor staurosporine, the PKA–PKI complex remained stable up to a CE of 50 V, confirming the strength of the inhibitory (noncovalent) PKA/PKI interaction, and consistent with the picomolar affinity of PKI for PKA [34,35,57].

### The PKI-binding conformer of PKAc has enhanced structural stability

The preferential binding of PKI to the more compact PKAc conformer led us to question whether there was a difference in the structural stability of these species. The non-PKI-bound PKAc remaining in the presence of excess PKI, PKA(PKI), was thus subjected to CIU (Figure 4) and compared with the unfolding profiles of WT, R133A and K72H PKAc (Figure 2). The CIU profile of PKA(PKI) was very similar to that observed for R133A; both demonstrate a significant reduction in gas-phase stability compared with WT PKAc, with collision-mediated unfolding starting at ∼32 V. These data suggest that it is the conformation, rather than phosphorylation status, of PKAc that regulates its structural stability and ability to bind to PKI.

### PKI disrupts hetero-oligomers of PKA catalytic and regulatory subunits

**Figure 5.**
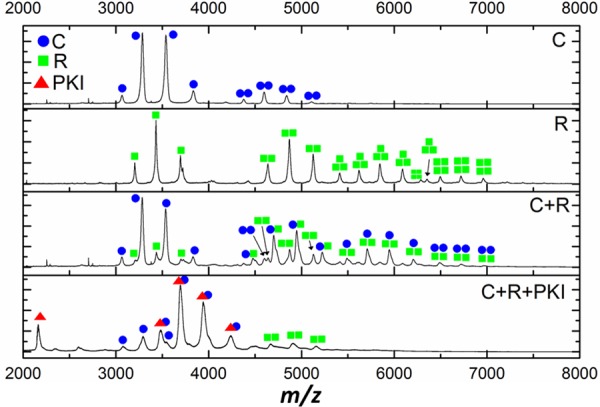
PKI disrupts non-covalent complexes between PKAc and RII. Native ESI mass spectra of WT PKAc (C, blue dots), PKA regulatory subunit RIIα (R, green squares) and equimolar ratios of the two (C + R) in the absence or presence of PKI proteins (red triangles).

To examine the disruptive effects of PKI on the physiological (catalytically inactive) hetero-tetrameric R_2_C_2_ complex of PKAc and PKA RIIα regulatory subunits, we undertook a series of novel MS and biochemical analyses. As shown in Figure 5, under native ESI conditions, we clearly observe monomers and homodimers of PKAc, monomers, homodimers, trimers and tetramers of RIIα, and various combinations of multimers after reconstitution of the PKA holoenzyme from individually purified components at a 1:1 ratio. Importantly, we can readily detect the physiological R_2_C_2_ holoenzyme complex. Dramatically, titration of equimolar PKI completely ablates observation of the R_2_C_2_ complex, additionally disrupting R_2_C, CR and C_2_ complexes, with preferential formation of the PKAc:PKI heterodimer. Disruption of PKAc, RIIα complexes by PKI, and formation of a PKAc:PKI heterodimer was also confirmed by SEC, the standard biochemical method for PKA complex analysis (Supplementary Figure S5). Consistent with previously published data with the RI subunit [62], there was no evidence for holoenzyme complex formation and RIIα binding with either the inactive K72H or the catalytically active R133A [57] variants either by SEC (Supplementary Figure S5) or native MS analysis.

### Binding of small molecules to PKAc decreases conformational flexibility

**Figure 6.**
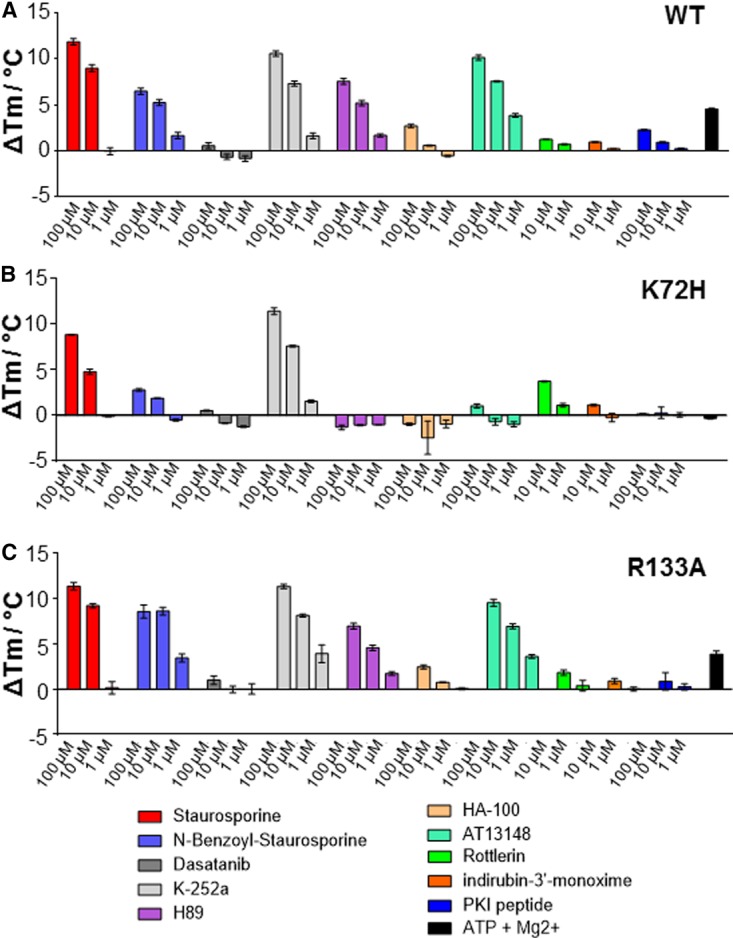
Binding of small-molecule kinase inhibitors to PKAc as determined by DSF. Thermal stability of WT (**A**), K72H (**B**) or R133A (**C**) PKAc (5 μM) was assessed as a function of inhibitor binding at the indicated concentrations. Mean Δ*T*_m_ values ± SD (*n* = 2) are shown.

The reversible binding of small-molecule ligands has revolutionized the study of protein kinases and is a powerful strategy for inhibiting (or activating) the catalytic activity of protein kinases *in vitro* and *in vivo* [10]*.* Using DSF, we assessed the effect of a panel of small-molecule PKA inhibitors on PKAc thermostability (Figure 6). We also evaluated compound binding to the catalytically inactive K72H variant, which we demonstrated (Figures 3 and 4) could not interact with nucleotides. Remarkably, many ATP mimetics known to target the PKAc ATP site markedly increased thermostability of K72H PKAc. Staurosporine, an indolocarbazole that is a pan-protein kinase inhibitor, stabilizes PKAc WT and K72H equally well, as do the closely related (but more specific) compounds *N*-benzoyl staurosporine and K-252a. In contrast, and in a similar fashion to the pseudosubstrate PKI full-length protein or PKI (5–23) peptide, the AGC kinase sulfonylisoquinoline inhibitors H89, HA-100 and the oral pan AGC inhibitor AT13148 bind only to WT and R133A PKAc under these conditions, and not the catalytically inactive (unphosphorylated) K72H PKAc, suggesting conformer-specific selection by specific ligand classes. This raises the question as to whether catalytic activity is a useful indication of the potential ability of kinase domains to bind ATP-competitive molecules *in vitro* and in cells and whether the physiological effects of inhibitors may be mediated in a compound-specific manner through interactions with ‘inactive’ conformers found in both canonical protein kinases and the multiple different classes of protein pseudokinase [70].

**Figure 7.**
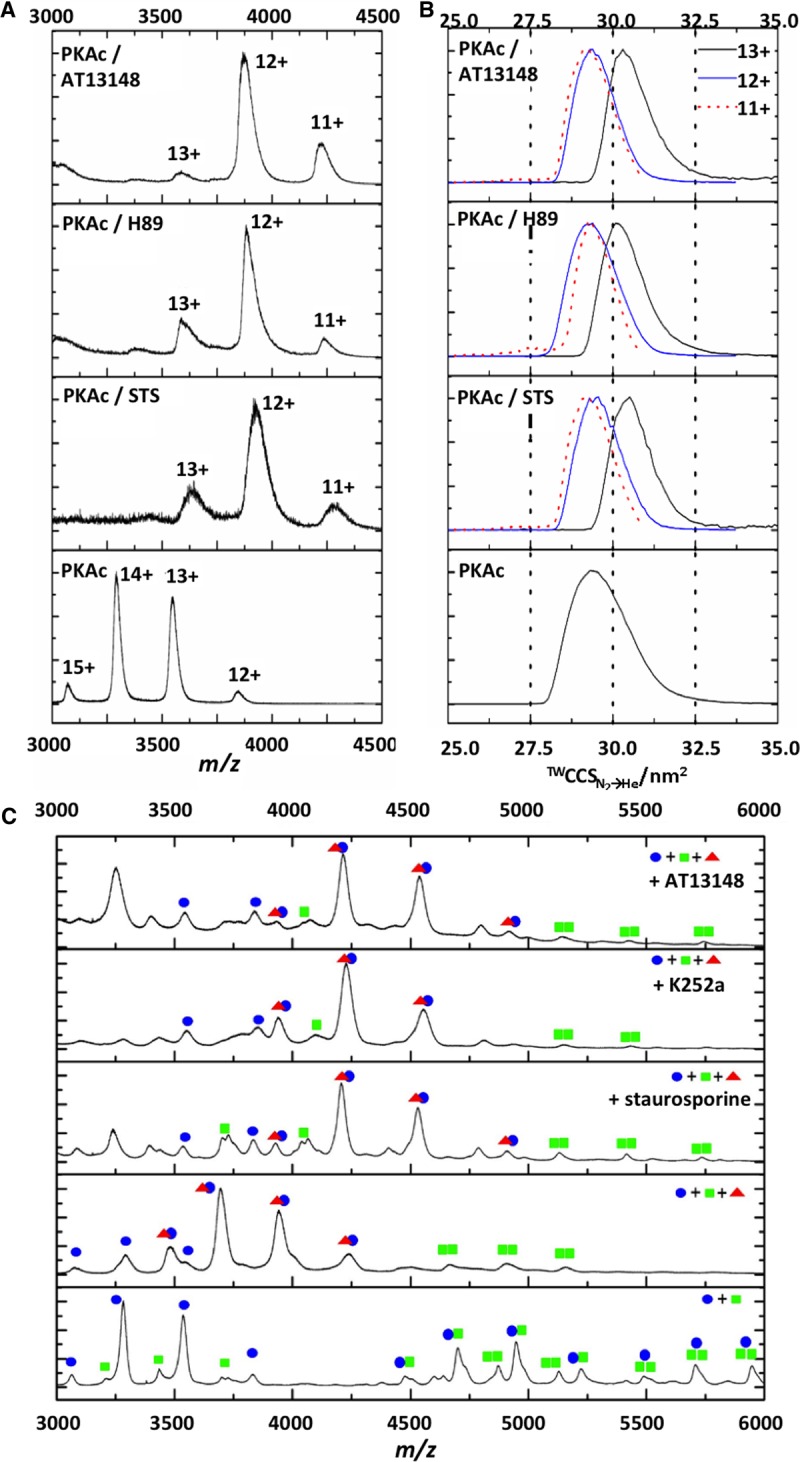
Binding of small-molecule inhibitors to PKAc and effect on disruption of PKA holoenzyme by PKI. Native ESI mass spectra (**A**) and ^TW^CCS_N2→He_ distributions (**B**) acquired in the presence of DMSO vehicle or with 10-fold molar excess of staurosporine (STS), H89 or AT13148. CCSD are presented for [M+11H]^11+^ (red dotted line), [M+12H]^12+^ (blue line) and [M+13H]^13+^ (black line). (**C**) Equimolar ratios of WT PKAc (C, blue dots) and PKA RIIα (R, green squares) were preincubated with vehicle control, STS, K252a or AT13148, prior to the addition of equipmolar PKI (red triangles). Native ESI spectra are annotated with identified complexes.

To corroborate our DSF assays, binding of the PKA inhibitor H89, the oral multi-AGC kinase inhibitor AT13148 (which is in phase I clinical trials) [71] and staurosporine [72] was confirmed by native ESI-MS (Figure 7 and Supplementary Figure S6).

Given our discovery of preferential binding of PKI to a specific PKA conformer, we also assessed the effect of these small molecules on PKAc conformation by IM–MS. Interestingly, there was a reduction in the charge states observed upon incubation with all three inhibitors, and a slight reduction in CSD, potentially suggesting a more ordered structure upon inhibitor binding. Inhibitor-dependent changes in CCSD for the same charge state could not thus be compared directly. Average Ω could be analyzed between the lowest two charge states for vehicle control (12+, 13+) and inhibitor bound (11+, 12+), as these were deemed to be most representative of the stable folded PKAc structures. All the inhibitor-bound forms of PKAc exhibited reduced conformational flexibility, with CCSD decreasing over 20% from 2.1 nm^2^ to <1.6 nm^2^. CCS also decreased upon inhibitor binding (in agreement with the reduction in CCSD), suggesting preferential adoption of a more compact PKAc conformer. Unlike PKI binding to PKAc, interaction with these inhibitors does not appear to be conformation-dependent, although binding did promote structural compaction with reduced conformational flexibility.

Given the indiscriminate binding of staurosporine and K252a to both WT and K72H PKAc, and formation of selective complexes between AT13148 and WT PKAc, we reasoned that there must be a difference in the binding mode of these small-molecule ligands that could potentially regulate interaction between members of the PKA signaling module. We therefore evaluated whether preincubation with a small molecule influenced PKI:PKA binding or PKI disruption of the PKA holoenzyme. Unlike PKI, staurosporine exhibited conformer-independent binding to PKAc, since no unbound catalytic subunit was observed (Supplementary Figure S7), consistent with our solution DSF data. Increasing the CE applied to the PKA:small molecule:PKI complex preferentially resulted in dissociation of the small molecule (∼55 V), leaving the stable PKA:PKI heterodimer. Interestingly, there was no discernible effect of PKI disruption of the PKA holoenzyme when it was performed in the presence of any ATP-competitive inhibitors, demonstrating independent binding mechanisms (Figure 7), which are entirely consistent with DSF analysis (Figure 4B).

## Conclusions

The complementary techniques of DSF and IM–MS are exploited here to examine structure, stability and binding of proteins and inhibitors to PKA signaling components *in vitro*. Even though PKA is used as a benchmark for studying other protein kinases, MS-based structural evaluation of the PKA complexes has not previously been reported to the best of our knowledge. MS studies previously evaluated ligand and inhibitor sensitivity of type I cGMP-dependent protein kinase (PKG) subunits [73,74]. However, PKG differs from PKA in that its regulation involves sensing of the cyclic GMP nucleotide within a single polypeptide. In the present paper, we report the first MS-based characterization of the PKA holoenzyme and explore heteromeric PKA complexes using native MS and IM–MS. By building on previous studies of PKA autophosphorylation after isolation from bacteria [25,26], we identify three novel sites of C-subunit autophosphorylation. Future studies will evaluate whether these sites are also phosphorylated in cells and *in vivo*, and whether they contribute to catalytic activity, and the formation or stability of PKA protein:protein complexes or both. We provide new evidence by IM–MS that PKAc exists in two primary conformations that are differentially permissive for ligand binding at the PKI (protein pseudosubstrate) site on the surface of the protein. Unfortunately, low MS resolution using ESI under native conditions prevents us from categorically determining if the phosphorylation status of the two C-subunit conformers are different. However, PKAc that has reduced (λPPase-treated) or completely lacks (K72H) phosphorylation preferentially exhibits an elongated conformation, similar to that of the elongated WT PKAc structure that does not bind to PKI. This predicts that these structural differences might, in part, be driven by altered phosphorylation status, similar to previous data that uncovered marked differences in structural stability between myristoylated and nonmyristoylated PKAc proteins [29,75]. However, our finding that the CCS value of normally phosphorylated R133A PKAc is identical with that of the larger (more elongated) WT PKAc conformer, and similar to that of unphosphorylated K72H, confirms that the structural diversity of PKAc is driven by factors in addition to the extent of phosphorylation. Modeling of R133A PKAc predicts an altered conformation, caused by ablation of the positive charge at Arg133 and loss of an electrostatic interaction with Glu230 [57]. Based on our work with R133A PKAc, we propose that this mutation can drive the phosphorylation-independent conformational differences observed by IM–MS. In this context, in the future, it will be interesting to compare these structural effects with those of stoichiometrically myristoylated PKAc, and a panel of PKAc phosphorylation site mutants, including the three novel sites identified in our *in vitro* study, whose cellular relevance is currently unknown.

Interestingly, we find that the smaller (less elongated) of the two PKAc conformers preferentially binds the pseudosubstrate inhibitor protein PKI, a pM affinity regulatory subunit with complex biological roles [37]. Consistent with this observation, the smaller conformer was absent upon IM–MS analysis of the K72H and R133A PKAc mutants, which are significantly impaired in their ability to bind PKI [21]. Distinct MS-unfolding profiles were observed for the non-PKI-binding form of PKA compared with the mixed conformer species. Of particular interest, the conformer incapable of binding PKI exhibited a markedly lower structural stability than the inhibitor-sensitive C-subunit population. Native MS analysis also reveals ternary formation of tetrameric PKA holoenzyme R_2_C_2_ complexes, as well as mixed R_2_C and RC multimers *in vitro*. Furthermore, we provide unambiguous experimental evidence that PKI directly disrupts the formation of all possible R:C complexes (likely at the R:C interface), confirming the exclusive binding of PKI to structurally permissive PKAc subunits, even in the presence of RIIα. The resultant (noncovalent) PKA:PKI complex is extremely stable, exhibiting a multistage unfolding profile with populations of discrete stable (partially unfolded) intermediate structures. It will be interesting to translate our findings to the cellular environment, where subcellular PKAc complexes with differential affinity for PKI could exist, with inhibitor affinity regulated as a function of PKAc phosphorylation or heterodimerization. In this regard, the analysis of PKI binding to nonphosphorylated, myristoylated or mutated PKAc, or PKAc complexes disrupted by cell-permeable AKAP ligands [76], will be of particular interest.

Based on careful MS analysis, kinase-dead (K72H) PKAc does not autophosphorylate when expressed in bacteria, probably due to an inability to bind nucleotides (either in the presence or absence of divalent metal cations), as confirmed using a DSF assay previously validated to detect binding of a variety of ligands to a panel of catalytically inactive and ATP binding-deficient pseudokinases [47]. Interestingly, the K72H mutant behaves as a potent ‘dominant negative’ *in vivo* when injected into oocytes [21], and retains the ability to bind to the ATP-competitive small-molecule inhibitors staurosporine and K-252a, but not isoquiolinesulfonyl PKAc inhibitors [77] or the clinical candidate AT13148. The ability of the type I inhibitor staurosporine to stabilize an ‘open’ (inactive) conformation of phosphorylated PKI-bound PKAc is known from X-ray analysis, and a potential induced van der Waals interaction with Lys 72 has been postulated [78]. However, our DSF analysis confirms that K72H PKAc still binds with high affinity to staurosporines. Interestingly, the preferential targeting of ‘type II’ kinase inhibitor classes to inactive kinase conformers of tyrosine kinases is well known and clinically relevant [43,51]. However, the propensity of inactive Ser/Thr kinases, exemplified by K72H PKAc (the present study) or multiple pseudokinases [47], to bind to different classes of kinase inhibitor is an important new area of research focus [70,79,80]. Our work demonstrates that, for an unphosphorylated K72H PKAc mutant, an inability to bind to ATP does not preclude binding to bisindole-based ATP-competitive inhibitors whose lack of specificity precludes their rational exploitation as chemical biology probe compounds [19]. In the absence of a crystal structure, it is challenging to explain why we are unable to detect K72H nucleotide binding using standard procedures [65], especially given previous findings [56,62]. However, Lys72 of PKA is reported to interact with the α- and β-phosphates of ATP and a critical C-helix Glu residue in the closed, active PKA structure [69]; mutation of the equivalent residue in (pseudo)kinases can ablate ATP binding that can be quantified by solution DSF [47,81]. We therefore speculate that should ATP still bind to K72H PKAc, its conformation must be markedly different from that of WT and R133A PKAc, but nonetheless remain responsive to thermal stabilization by some kinase inhibitors. All the kinase inhibitors evaluated in the present study are formally ATP-competitive (type I) kinase inhibitors, and our work has ramifications for studying cellular small-molecule ligand effects on phosphorylated or inactive kinases, predicting that, for PKAc, phosphorylation might also be able to indirectly control kinase:small-molecule interactions.

We conclude that analytical DSF complements IM–MS and CIU for interrogating ligand-binding and ligand-mediated conformational dynamics of PKA signaling components. These strategies are quantitative, permit evaluation of specific and nonspecific interactions, and enhance our understanding of the structural effects pertaining to ligand:protein interactions *in vitro*. Indeed, we provide new insights into the PKA holoenzyme complex, a signaling system that continues to inspire, half a century after its discovery.

## Abbreviations

AKAPs, A-kinase anchoring proteins; CCS, collision cross section; CCSD, CCS distribution; CD, circular dichroism; CE, collision energy; CIU, collision-induced unfolding; CSD, charge state distribution; DSF, differential scanning fluorimetry; EHSS, exact hard sphere scattering; GST, Glutathione-*S*-transferase; IM-MS, ion mobility–mass spectrometry; MS, mass spectrometry; NES, nuclear export signal; NMR, nuclear magnetic resonance; PKA, cAMP-dependent protein kinase; PKAc, PKA catalytic (C) subunits; PKI, heat-stable PKA inhibitor protein; PTM, post-translational modification; SEC, size-exclusion chromatography; TSA, thermostability assay; T-wave, travelling wave; WT, wild type; λPP, λ protein phosphatase.

## Author Contribution

The manuscript was written with the contributions of all authors. All authors have given approval to the final version of the manuscript.

## Funding

This work was supported by core funding from the Institute of Integrative Biology, University of Liverpool, the BBSRC [grant BB/L009501/1] and a BBSRC DTP PhD studentship (supporting S.F.).

## References

[1] Cohen P. (2001) The role of protein phosphorylation in human health and disease. The Sir Hans Krebs Medal Lecture. Eur. J. Biochem. 268, 5001–5010 doi:10.1046/j.0014-2956.2001.02473.x 11589691

[2] Uetrecht C., Rose R.J., van Duijn E., Lorenzen K. and Heck A.J. (2010) Ion mobility mass spectrometry of proteins and protein assemblies. Chem. Soc. Rev. 39, 1633–1655 doi:10.1039/B914002F 20419213

[3] Taylor S.S., Ilouz R., Zhang P. and Kornev A.P. (2012) Assembly of allosteric macromolecular switches: lessons from PKA. Nat. Rev. Mol. Cell Biol. 13, 646–658 doi:10.1038/nrm3432 22992589 PMC3985763

[4] Srivastava A.K., McDonald L.R., Cembran A., Kim J., Masterson L.R., McClendon C.L. et al. (2014) Synchronous opening and closing motions are essential for cAMP-dependent protein kinase A signaling. Structure 22, 1735–1743 doi:10.1016/j.str.2014.09.010 25458836 PMC4255147

[5] Kornev A.P. and Taylor S.S. (2015) Dynamics-driven allostery in protein kinases. Trends Biochem. Sci. 40, 628–647 doi:10.1016/j.tibs.2015.09.002 26481499 PMC4630092

[6] Harvey S.R., Porrini M., Stachl C., MacMillan D., Zinzalla G. and Barran P.E. (2012) Small-molecule inhibition of c-MYC:MAX leucine zipper formation is revealed by ion mobility mass spectrometry. J. Am. Chem. Soc. 134, 19384–19392 doi:10.1021/ja306519h 23106332

[7] Niu S., Rabuck J.N. and Ruotolo B.T. (2013) Ion mobility-mass spectrometry of intact protein–ligand complexes for pharmaceutical drug discovery and development. Curr. Opin. Chem. Biol. 17, 809–817 doi:10.1016/j.cbpa.2013.06.019 23856053

[8] Bastidas A.C., Deal M.S., Steichen J.M., Guo Y., Wu J. and Taylor S.S. (2013) Phosphoryl transfer by protein kinase A is captured in a crystal lattice. J. Am. Chem. Soc. 135, 4788–4798 doi:10.1021/ja312237q 23458248 PMC3663052

[9] Cohen P. and Alessi D.R. (2013) Kinase drug discovery — what's next in the field? ACS Chem. Biol. 8, 96–104 doi:10.1021/cb300610s 23276252 PMC4208300

[10] Fabbro D. (2015) 25 years of small molecular weight kinase inhibitors: potentials and limitations. Mol. Pharmacol. 87, 766–775 doi:10.1124/mol.114.095489 25549667

[11] Eyers P.A. and Murphy J.M. (2013) Dawn of the dead: protein pseudokinases signal new adventures in cell biology. Biochem. Soc. Trans. 41, 969–974 doi:10.1042/BST20130115 23863165

[12] Adams J.A. (2001) Kinetic and catalytic mechanisms of protein kinases. Chem. Rev. 101, 2271–2290 doi:10.1021/cr000230w 11749373

[13] Masterson L.R., Shi L., Metcalfe E., Gao J., Taylor S.S. and Veglia G. (2011) Dynamically committed, uncommitted, and quenched states encoded in protein kinase A revealed by NMR spectroscopy. Proc. Natl Acad. Sci. USA 108, 6969–6974 doi:10.1073/pnas.1102701108 21471451 PMC3084134

[14] Knight Z.A., Lin H. and Shokat K.M. (2010) Targeting the cancer kinome through polypharmacology. Nat. Rev. Cancer 10, 130–137 doi:10.1038/nrc2787 20094047 PMC2880454

[15] Walsh D.A., Perkins J.P. and Krebs E.G. (1968) An adenosine 3′,5′-monophosphate-dependant protein kinase from rabbit skeletal muscle. J. Biol. Chem. 243, 3763–3765 PMID: 4298072

[16] Langeberg L.K. and Scott J.D. (2015) Signalling scaffolds and local organization of cellular behaviour. Nat. Rev. Mol. Cell Biol. 16, 232–244 doi:10.1038/nrm3966 25785716 PMC4722875

[17] Pearce L.R., Komander D. and Alessi D.R. (2010) The nuts and bolts of AGC protein kinases. Nat. Rev. Mol. Cell Biol. 11, 9–22 doi:10.1038/nrm2822 20027184

[18] Koide K., Bunnage M.E., Gomez Paloma L., Kanter J.R., Taylor S.S., Brunton L.L. et al. (1995) Molecular design and biological activity of potent and selective protein kinase inhibitors related to balanol. Chem. Biol. 2, 601–608 doi:10.1016/1074-5521(95)90124-8 9383464

[19] Davies S.P., Reddy H., Caivano M. and Cohen P. (2000) Specificity and mechanism of action of some commonly used protein kinase inhibitors. Biochem. J. 351, 95–105 doi:10.1042/bj3510095 10998351 PMC1221339

[20] Masterson L.R., Mascioni A., Traaseth N.J., Taylor S.S. and Veglia G. (2008) Allosteric cooperativity in protein kinase A. Proc. Natl Acad. Sci. USA. 105, 506–511 doi:10.1073/pnas.0709214104 18178622 PMC2206566

[21] Eyers P.A., Liu J., Hayashi N.R., Lewellyn A.L., Gautier J. and Maller J.L. (2005) Regulation of the G_2_/M transition in *Xenopus* oocytes by the cAMP-dependent protein kinase. J. Biol. Chem. 280, 24339–24346 doi:10.1074/jbc.M412442200 15860459

[22] Adams S.R., Harootunian A.T., Buechler Y.J., Taylor S.S. and Tsien R.Y. (1991) Fluorescence ratio imaging of cyclic AMP in single cells. Nature 349, 694–697 doi:10.1038/349694a0 1847505

[23] Maller J.L. and Krebs E.G. (1977) Progesterone-stimulated meiotic cell division in Xenopus oocytes. Induction by regulatory subunit and inhibition by catalytic subunit of adenosine 3′:5′-monophosphate-dependent protein kinase. J. Biol. Chem. 252, 1712–1718 PMID: 190238

[24] Zhang P., Kornev A.P., Wu J. and Taylor S.S. (2015) Discovery of allostery in PKA signaling. Biophys. Rev. 7, 227–238 doi:10.1007/s12551-015-0170-x 26097522 PMC4469037

[25] Yonemoto W., McGlone M.L., Grant B. and Taylor S.S. (1997) Autophosphorylation of the catalytic subunit of cAMP-dependent protein kinase in *Escherichia coli*. Protein Eng. Des. Select. 10, 915–925 doi:10.1093/protein/10.8.915 9415441

[26] Seidler J., Adal M., Kübler D., Bossemeyer D. and Lehmann W.D. (2009) Analysis of autophosphorylation sites in the recombinant catalytic subunit alpha of cAMP-dependent kinase by nano-UPLC-ESI-MS/MS. Anal. Bioanal. Chem. 395, 1713–1720 doi:10.1007/s00216-009-2932-4 19590856

[27] Thinon E., Serwa R.A., Broncel M., Brannigan J.A., Brassat U., Wright M.H. et al. (2014) Global profiling of co- and post-translationally N-myristoylated proteomes in human cells. Nat. Commun. 5, 4919 doi:10.1038/ncomms5919 25255805 PMC4200515

[28] Zhang P., Ye F., Bastidas A.C., Kornev A.P., Wu J., Ginsberg M.H. et al. (2015) An isoform-specific myristylation switch targets type II PKA holoenzymes to membranes. Structure 23, 1563–1572 doi:10.1016/j.str.2015.07.007 26278174 PMC4558360

[29] Bastidas A.C., Deal M.S., Steichen J.M., Keshwani M.M., Guo Y. and Taylor S.S. (2012) Role of N-terminal myristylation in the structure and regulation of cAMP-dependent protein kinase. J. Mol. Biol. 422, 215–229 doi:10.1016/j.jmb.2012.05.021 22617327 PMC3597442

[30] Carnegie G.K., Means C.K. and Scott J.D. (2009) A-kinase anchoring proteins: from protein complexes to physiology and disease. IUBMB Life 61, 394–406 doi:10.1002/iub.168 19319965 PMC2682206

[31] Erlichman J., Rubin C.S. and Rosen O.M. (1973) Physical properties of a purified cyclic adenosine 3′:5′-monophosphate-dependent protein kinase from bovine heart muscle. J. Biol. Chem. 248, 7607–7609 4355589

[32] Rubin C.S., Erlichman J. and Rosen O.M. (1972) Molecular forms and subunit composition of a cyclic adenosine 3′,5′-monophosphate-dependent protein kinase purified from bovine heart muscle. J. Biol. Chem. 247, 36–44 4336043

[33] Guo Y., Wilderman A., Zhang L., Taylor S.S. and Insel P.A. (2012) Quantitative proteomics analysis of the cAMP/protein kinase A signaling pathway. Biochemistry 51, 9323–9332 doi:10.1021/bi301282k 23110364 PMC3503394

[34] Walsh D.A., Ashby C.D., Gonzalez C., Calkins D., Fischer E.H. and Krebs E.G. (1971) Purification and characterization of a protein inhibitor of adenosine 3′,5′-monophosphate-dependent protein kinases. J. Biol. Chem. 246, 1977–1985 4324557

[35] Scott J.D., Glaccum M.B., Fischer E.H. and Krebs E.G. (1986) Primary-structure requirements for inhibition by the heat-stable inhibitor of the cAMP-dependent protein kinase. Proc. Natl Acad. Sci. USA 83, 1613–1616 doi:10.1073/pnas.83.6.1613 3456605 PMC323133

[36] Scott J.D., Fischer E.H., Takio K., Demaille J.G. and Krebs E.G. (1985) Amino acid sequence of the heat-stable inhibitor of the cAMP-dependent protein kinase from rabbit skeletal muscle. Proc. Natl Acad. Sci. USA 82, 5732–5736 doi:10.1073/pnas.82.17.5732 3898070 PMC390626

[37] Kawakami M. and Nakanishi N. (2001) The role of an endogenous PKA inhibitor, PKIalpha, in organizing left-right axis formation. Development 128, 2509–2515 PMID: 11493567 10.1242/dev.128.13.2509

[38] Iglesias-Bartolome R., Torres D., Marone R., Feng X., Martin D., Simaan M. et al. (2015) Inactivation of a Gαs–PKA tumour suppressor pathway in skin stem cells initiates basal-cell carcinogenesis. Nat. Cell Biol. 17, 793–803 doi:10.1038/ncb3164 25961504 PMC4449815

[39] Wen W., Meinkotht J.L., Tsien R.Y. and Taylor S.S. (1995) Identification of a signal for rapid export of proteins from the nucleus. Cell 82, 463–473 doi:10.1016/0092-8674(95)90435-2 7634336

[40] Masterson L.R., Cheng C., Yu T., Tonelli M., Kornev A., Taylor S.S. et al. (2010) Dynamics connect substrate recognition to catalysis in protein kinase A. Nat. Chem. Biol. 6, 821–828 doi:10.1038/nchembio.452 20890288 PMC3487389

[41] Kim J., Li G., Walters M.A., Taylor S.S. and Veglia G. (2016) Uncoupling catalytic and binding functions in the cyclic AMP-dependent protein kinase A. Structure 24, 353–363 doi:10.1016/j.str.2015.11.016 26833386 PMC4775281

[42] De Moliner E., Brown N.R. and Johnson L.N. (2003) Alternative binding modes of an inhibitor to two different kinases. Eur. J. Biochem. 270, 3174–3181 doi:10.1046/j.1432-1033.2003.03697.x 12869192

[43] Nagar B., Bornmann W.G., Pellicena P., Schindler T., Veach D.R., Miller W.T. et al. (2002) Crystal structures of the kinase domain of c-Abl in complex with the small molecule inhibitors PD173955 and imatinib (STI-571). Cancer Res. 62, 4236–4243 PMID: 12154025

[44] Kornev A.P., Taylor S.S. and Ten Eyck L.F. (2008) A helix scaffold for the assembly of active protein kinases. Proc. Natl Acad. Sci. USA 105, 14377–14382 doi:10.1073/pnas.0807988105 18787129 PMC2533684

[45] McClendon C.L., Kornev A.P., Gilson M.K. and Taylor S.S. (2014) Dynamic architecture of a protein kinase. Proc. Natl Acad. Sci. USA 111, E4623–E4631 doi:10.1073/pnas.1418402111 25319261 PMC4217441

[46] Mohanty S., Oruganty K., Kwon A., Byrne D.P., Ferries S., Ruan Z. et al. (2016) Hydrophobic core variations provide a structural framework for tyrosine kinase evolution and functional specialization. PLoS Genet. 12, e1005885 doi:10.1371/journal.pgen.1005885 26925779 PMC4771162

[47] Murphy J.M., Zhang Q., Young S.N., Reese M.L., Bailey F.P., Eyers P.A. et al. (2014) A robust methodology to subclassify pseudokinases based on their nucleotide-binding properties. Biochem. J. 457, 323–334 doi:10.1042/BJ20131174 24107129 PMC5679212

[48] Lanucara F., Holman S.W., Gray C.J. and Eyers C.E. (2014) The power of ion mobility-mass spectrometry for structural characterization and the study of conformational dynamics. Nat. Chem. 6, 281–294 doi:10.1038/nchem.1889 24651194

[49] Niesen F.H., Berglund H. and Vedadi M. (2007) The use of differential scanning fluorimetry to detect ligand interactions that promote protein stability. Nat. Protoc. 2, 2212–2221 doi:10.1038/nprot.2007.321 17853878

[50] Savitski M.M., Reinhard F.B.M., Franken H., Werner T., Savitski M.F., Eberhard D. et al. (2014) Tracking cancer drugs in living cells by thermal profiling of the proteome. Science 346, 1255784 doi:10.1126/science.1255784 25278616

[51] Rabuck J.N., Hyung S.-J., Ko K.S., Fox C.C., Soellner M.B. and Ruotolo B.T. (2013) Activation state-selective kinase inhibitor assay based on ion mobility-mass spectrometry. Anal. Chem. 85, 6995–7002 doi:10.1021/ac4012655 23845095 PMC3784979

[52] Hyung S.-J., Robinson C.V. and Ruotolo B.T. (2009) Gas-phase unfolding and disassembly reveals stability differences in ligand-bound multiprotein complexes. Chem. Biol. 16, 382–390 doi:10.1016/j.chembiol.2009.02.008 19389624

[53] Smith F.D., Reichow S.L., Esseltine J.L., Shi D., Langeberg L.K., Scott J.D. et al. (2013) Intrinsic disorder within an AKAP-protein kinase A complex guides local substrate phosphorylation. eLife 2, e01319 http://dx.doi.org/10.7554/eLife.01319 24192038 10.7554/eLife.01319 PMC3814001

[54] Ruotolo B.T., Benesch J.L., Sandercock A.M., Hyung S.-J. and Robinson C.V. (2008) Ion mobility-mass spectrometry analysis of large protein complexes. Nat. Protoc. 3, 1139–1152 doi:10.1038/nprot.2008.78 18600219

[55] Shvartsburg A.A. and Jarrold M.F. (1996) An exact hard-spheres scattering model for the mobilities of polyatomic ions. Chem. Phys. Lett. 261, 86–91 doi:10.1016/0009-2614(96)00941-4

[56] Iyer G.H., Garrod S.V., Woods V.L. Jr and Taylor S.S. (2005) Catalytic independent functions of a protein kinase as revealed by a kinase-dead mutant: study of the Lys72His mutant of cAMP-dependent kinase. J. Mol. Biol. 351, 1110–1122 doi:10.1016/j.jmb.2005.06.011 16054648

[57] Wen W. and Taylor S.S. (1994) High affinity binding of the heat-stable protein kinase inhibitor to the catalytic subunit of cAMP-dependent protein kinase is selectively abolished by mutation of Arg133. J. Biol. Chem. 269, 8423–8430 PMID: 8132568

[58] Keshwani M.M., Klammt C., von Daake S., Ma Y., Kornev A.P., Choe S. et al. (2012) Cotranslational cis-phosphorylation of the COOH-terminal tail is a key priming step in the maturation of cAMP-dependent protein kinase. Proc. Natl Acad. Sci. USA 109, E1221–E1229 doi:10.1073/pnas.1202741109 22493239 PMC3356610

[59] Steichen J.M., Iyer G.H., Li S., Saldanha S.A., Deal M.S., Woods V.L. Jr et al. (2010) Global consequences of activation loop phosphorylation on protein kinase A. J. Biol. Chem. 285, 3825–3832 doi:10.1074/jbc.M109.061820 19965870 PMC2823524

[60] Haydon C.E., Eyers P.A., Aveline-Wolf L.D., Resing K.A., Maller J.L. and Ahn N.G. (2003) Identification of novel phosphorylation sites on Xenopus laevis Aurora A and analysis of phosphopeptide enrichment by immobilized metal-affinity chromatography. Mol. Cell. Proteomics 2, 1055–1067 doi:10.1074/mcp.M300054-MCP200 12885952

[61] Toner-Webb J., van Patten S.M., Walsh D.A. and Taylor S.S. (1992) Autophosphorylation of the catalytic subunit of cAMP-dependent protein kinase. J. Biol. Chem. 267, 25174–25180 PMID: 1460017

[62] Iyer G.H., Moore M.J. and Taylor S.S. (2005) Consequences of lysine 72 mutation on the phosphorylation and activation state of cAMP-dependent kinase. J. Biol. Chem. 280, 8800–8807 doi:10.1074/jbc.M407586200 15618230

[63] Beveridge R., Chappuis Q., Macphee C. and Barran P. (2013) Mass spectrometry methods for intrinsically disordered proteins. Analyst 138, 32–42 doi:10.1039/C2AN35665A 23108160

[64] Hopper J.T. and Oldham N.J. (2009) Collision induced unfolding of protein ions in the gas phase studied by ion mobility-mass spectrometry: the effect of ligand binding on conformational stability. J. Am. Soc. Mass Spectr. 20, 1851–1858 doi:10.1016/j.jasms.2009.06.010 19643633

[65] Herberg F.W., Doyle M.L., Cox S. and Taylor S.S. (1999) Dissection of the nucleotide and metal–phosphate binding sites in cAMP-dependent protein kinase. Biochemistry 38, 6352–6360 doi:10.1021/bi982672w 10320366

[66] Knape M.J., Ahuja L.G., Bertinetti D., Burghardt N.C.G., Zimmermann B., Taylor S.S. et al. (2015) Divalent metal ions Mg^2+^ and Ca^2+^ have distinct effects on protein kinase A activity and regulation. ACS Chem. Biol. 10, 2303–2315 doi:10.1021/acschembio.5b00271 26200257 PMC4714867

[67] Akamine P., Madhusudan, Wu J., Xuong N.H., Ten Eyck L.F. and Taylor S.S. (2003) Dynamic features of cAMP-dependent protein kinase revealed by apoenzyme crystal structure. J. Mol. Biol. 327, 159–171 PMID: 12614615 10.1016/s0022-2836(02)01446-8

[68] Knighton D.R., Bell S.M., Zheng J., Ten Eyck L.F., Xuong N.H., Taylor S.S. et al. (1993) 2.0 A refined crystal structure of the catalytic subunit of cAMP-dependent protein kinase complexed with a peptide inhibitor and detergent. Acta Crystallogr. D Biol. Crystallogr. 49, 357–361 doi:10.1107/S0907444993000502 15299526

[69] Knighton D., Zheng J., Ten Eyck L., Ashford V., Xuong N., Taylor S. et al. (1991) Crystal structure of the catalytic subunit of cyclic adenosine monophosphate-dependent protein kinase. Science 253, 407–414 doi:10.1126/science.1862342 1862342

[70] Reiterer V., Eyers P.A. and Farhan H. (2014) Day of the dead: pseudokinases and pseudophosphatases in physiology and disease. Trends Cell Biol. 24, 489–505 doi:10.1016/j.tcb.2014.03.008 24818526

[71] Yap T.A., Walton M.I., Grimshaw K.M., te Poele R.H., Eve P.D., Valenti M.R. et al. (2012) AT13148 is a novel, oral multi-AGC kinase inhibitor with potent pharmacodynamic and antitumor activity. Clin. Cancer Res. 18, 3912–3923 doi:10.1158/1078-0432.CCR-11-3313 22781553

[72] Tamaoki T., Nomoto H., Takahashi I., Kato Y., Morimoto M. and Tomita F. (1986) Staurosporine, a potent inhibitor of phospholipidCa++ dependent protein kinase. Biochem. Biophys. Res. Commun. 135, 397–402 doi:10.1016/0006-291X(86)90008-2 3457562

[73] Pinkse M.W., Heck A.J., Rumpel K. and Pullen F. (2004) Probing noncovalent protein—ligand interactions of the cGMP-dependent protein kinase using electrospray ionization time of flight mass spectrometry. J. Am. Soc. Mass Spectr. 15, 1392–1399 doi:10.1016/j.jasms.2004.06.015 15465351

[74] Pinkse M.W.H., Rijkers D.T.S., Dostmann W.R. and Heck A.J.R. (2009) Mode of action of cGMP-dependent protein kinase-specific inhibitors probed by photoaffinity cross-linking mass spectrometry. J. Biol. Chem. 284, 16354–16368 doi:10.1074/jbc.M808521200 19369251 PMC2713552

[75] Yonemoto W., McGlone M.L. and Taylor S.S. (1993) N-myristylation of the catalytic subunit of cAMP-dependent protein kinase conveys structural stability. J. Biol. Chem. 268, 2348–2352 PMID: 8428909

[76] Wang Y., Ho T.G., Franz E., Hermann J.S., Smith F.D., Hehnly H. et al. (2015) PKA-type I selective constrained peptide disruptors of AKAP complexes. ACS Chem. Biol. 10, 1502–1510 doi:10.1021/acschembio.5b00009 25765284 PMC4475429

[77] Engh R.A., Girod A., Kinzel V., Huber R. and Bossemeyer D. (1996) Crystal structures of catalytic subunit of cAMP-dependent protein kinase in complex with isoquinolinesulfonyl protein kinase inhibitors H7, H8, and H89. Structural implications for selectivity. J. Biol. Chem. 271, 26157–26164 doi:10.1074/jbc.271.42.26157 8824261

[78] Prade L., Engh R.A., Girod A., Kinzel V., Huber R. and Bossemeyer D. (1997) Staurosporine-induced conformational changes of cAMP-dependent protein kinase catalytic subunit explain inhibitory potential. Structure 5, 1627–1637 doi:10.1016/S0969-2126(97)00310-9 9438863

[79] Foulkes D.M., Byrne D.P., Bailey F.P. and Eyers P.A. (2015) Tribbles pseudokinases: novel targets for chemical biology and drug discovery? Biochem. Soc. Trans. 43, 1095–1103 doi:10.1042/BST20150109 26517930

[80] Kung J.E. and Jura N. (2016) Structural basis for the non-catalytic functions of protein kinases. Structure 24, 7–24 doi:10.1016/j.str.2015.10.020 26745528 PMC4706642

[81] Bailey F.P., Byrne D.P., Oruganty K., Eyers C.E., Novotny C.J., Shokat K.M. et al. (2015) The Tribbles 2 (TRB2) pseudokinase binds to ATP and autophosphorylates in a metal-independent manner. Biochem. J. 467, 47–62 doi:10.1042/BJ20141441 25583260 PMC4844368

